# Effect of Aqueous and Ethyl Acetate Fractions of *Ziziphus jujuba* Mill Extract on Cardiovascular Responses in Hypertensive Rats

**DOI:** 10.21315/mjms2020.27.3.5

**Published:** 2020-06-30

**Authors:** Reza Mohebbati, Yasamin Kamkar-Del, Mohammad Naser Shafei

**Affiliations:** 1 Department of Physiology, Faculty of Medicine, Mashhad University of Medical Sciences, Mashhad, Iran; 2 Neurogenic Inflammation Research Center, Mashhad University of Medical Sciences, Mashhad, Iran

**Keywords:** Ziziphus jujuba Mill, hypertension, aqueous fraction, ethyl acetate fraction, blood pressure

## Abstract

**Background:**

*Ziziphus jujuba Mill* (ZJ) is a plant with anti-hypertensive property. In this regard, the present study investigated the effect of aqueous and ethyl acetate fractions of ZJ extract on acute hypertension (HTN) induced by nitro-L-arginine methyl ester (L-NAME).

**Methods:**

The current study was carried on 49 hypertensive rats divided into seven groups, including i) control; ii) L-NAME (10 mg/kg); iii) sodium nitroprusside (SNP) (50 μg/kg) plus L-NAME; iv and v) aqueous fraction of ZJ (150 mg/kg and 300 mg/kg) plus L-NAME; vi) and vii) ethyl acetate fractions of ZJ (150 mg/kg and 300 mg/kg) plus L-NAME. The rats were orally treated with both fractions for four weeks and received intravenous L-NAME on the 28th day. The mean arterial pressure (MAP), systolic blood pressure (SBP) and heart rate (HR) of the rats were recorded then maximal changes (Δ) of MAP, SBP and HR were calculated and compared with changes of control and L-NAME.

**Results:**

According to the obtained results of the present study, it was shown that the administration of L-NAME significantly increased ΔMAP, ΔSBP and ΔHR, and these effects were significantly attenuated by administration of SNP. The pre-treatment with both doses (150 mg/kg and 300 mg/kg) of aqueous and ethyl acetate fractions could significantly reduce cardiovascular responses induced by L-NAME that comparable with SNP. However, a lower dose of aqueous fractions and higher dose of ethyl acetate fractions were reported with stronger effects.

**Conclusion:**

The results of the current study showed that both the aqueous and ethyl acetate fractions of ZJ through the effect on nitric oxide system can prevent the development of HTN induced by L-NAME.

## Introduction

The endothelium is an important regulator of vascular homeostasis. One of the most important functions of endothelium is the adjustment and modulation of the vascular tone mainly by the release of vasodilators, such as nitric oxide (NO) and prostacyclin (1). In endothelium, NO is produced from L-arginine by endothelial nitric oxide synthase (eNOS) activity and plays a protective effect on the cardiovascular system. For example, endothelial dysfunction via the reduction of NO bioavailability results in some vascular problems, such as hypertension (HTN) (2). Furthermore, NOS inhibitors, including nitro-L-arginine methyl ester (L-NAME), induce HTN in animal models (3). Therefore, factors that increase NO bioavailability can help with the improvement of HTN. Currently, NO enhancers are also considered among the treatments of HTN (4).

According to the evidence, the anti-hypertensive effects of medicinal plants are partly mediated by an effect on NO. One of these plants is *Ziziphus jujuba Mill* (ZJ) named *annab* in Iran. The ZJ is a member of the Rhamnaceae family with numerous functions in traditional and classical medicine (5). Effective constituents, such as phenolic compounds, flavonoids, polysaccharides and vitamins (e.g. C and A), have been indicated in this plant (6, 7). Pharmacological effects, such as antioxidant (8), anti-inflammatory (9) and anti-cancer (10), as well as other protective properties of ZJ, have been shown attributed to the above-mentioned compounds. There has also been a report on the anti-hypertensive effect of ZJ. The anti-hypertensive effect of ZJ extract is due to the presence of antioxidants and polyphenols (11, 12).

In a recent study, it was also indicated that the hydroalcoholic extract of ZJ ameliorates cardiovascular parameters in HTN induced by L-NAME (13) that confirm the effect of ZJ extract on NO. In addition, the hydroalcoholic extract is a total extract and the solubility of compounds is different based on polarity. With this background in mind, the present study evaluated the effect of the two aqueous (i.e., polar) and ethyl acetate (i.e., semi-polar) fractions of ZJ on acute HTN induced by L-NAME to identify the effectiveness of the compounds in HTN induced by NO deficiency.

## Methods

### Fraction Preparation

The fruits of ZJ were provided from a herb store in Birjand, Iran and identified by botanists in the Herbarium of the Pharmacy School in Mashhad University of Medical Sciences, Mashhad, Iran (voucher no.: 13246). About 100 g of ZJ fruit was powdered, ground and then macerated in 1 L of 70% ethanol and shacked for three days at a temperature of 37 ºC. After that, the mixture was filtered using filters of different sizes. The solvent was evaporated by an oven at 40 ºC (13, 14). For fraction preparation, 50 g of ZJ extract was dissolved in 200 mL of distilled water. The solution was poured into the funnel decanter and the soluble material was isolated three times by adding 50 mL of ethyl acetate. The remaining solution in the funnel was an aqueous fraction. Then, both fractions were placed in the oven to remove the solvent (15).

### Cannulation of Artery and Recording of Cardiovascular Parameters

A total of 49 Wistar male rats purchased from the Animal Laboratory of Mashhad University of Medical Sciences were kept under the standard conditions in the animal room. The animals were anesthetised with sodium thiopental (60 mg/kg intraperitoneal [ip]) (16). Subsequently, the left femoral artery was cannulated with a 22-gauge angiocatheter filled with heparinised saline (50 U/mL). Then, the angiocatheter was connected to a blood pressure transducer (AD Instruments, Australia). Furthermore, blood pressure (BP) and heart rate (HR) were continuously recorded by a PowerLab System (AD Instruments, Australia) (17).

### Drugs

The drugs, including sodium thiopental, L-NAME and sodium nitroprusside (SNP), were provided by Sigma Pharmaceuticals in the USA. In this study, all the medications were dissolved in saline.

### Animal Groups

The rats were randomly divided into the seven following groups:

- Control (*n* = 7): The rats received intravenous (iv) saline;
- L-NAME (*n* = 7): The rats received L-NAME (10 mg/kg, iv);
- SNP (*n* = 7): The rats received SNP (50 μg/ kg, iv) and L-NAME after 10 min;
- Aqueous fractions of ZJ (150 mg/kg and 300 mg/kg) (*n* = 7; *n* = 7): The rats received 150 mg/kg and 300 mg/kg of aqueous fraction of ZJ orally for four weeks and L-NAME on the experiment day (the 28th day);
- Ethyl acetate fraction of ZJ (150 mg/kg and 300 mg/kg) (*n* = 7; *n* = 7): The rats received 150 mg/kg and 300 mg/kg doses of ethyl acetate fraction of ZJ according to the previous protocol.

### Experimental Protocol

The rats in the L-NAME received 10 mg/kg of L-NAME. In the SNP group, the animals firstly received 50 μg/kg dose of SNP and L-NAME after 10 min (18, 19). In the fraction groups, the rats received 150 mg/kg and 300 mg/kg doses of ZJ fractions (20) orally for four weeks. On the experiment day (i.e. the 28th day), the L-NAME was injected after the cannulation of the artery. In all groups, systolic blood pressure (SBP), mean arterial pressure (MAP) and HR were recorded throughout the experimental period. The injection of L-NAME and SNP was conducted via the vein of the tail.

### Data Analysis

The changes (Δ) of MAP, SBP and HR values were calculated and expressed as mean ± standard error of the mean. Statistical analysis was carried out by one-way analysis of variance followed by the Tukey’s post hoc test. A *P*-value of less than 0.05 was considered statistically significant.

## Results

### Effects of L-NAME and SNP on Cardiovascular Responses

**Figure 1:**
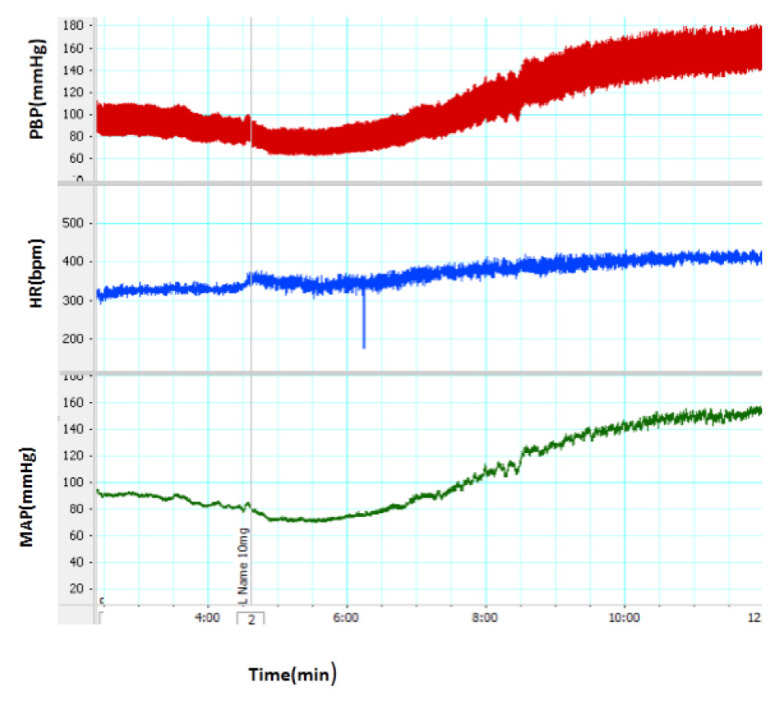
A recorded sample of cardiovascular responses induced by intravenous administration of L-NAME. The line indicates the time of injection

**Figure 2:**
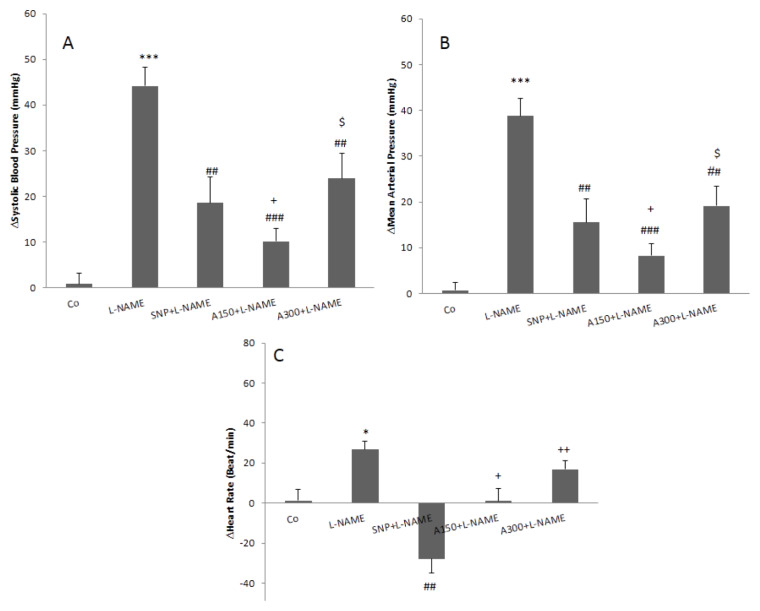
Effects of aqueous fractions of the ZJ extract on ΔSBP (A), ΔMAP (B) and ΔHR(C) in L-NAME hypertensive rats. Two doses of the fraction (150 mg/kg and 300 mg/kg) (A150 and A300) administrated orally for four weeks then L-NAME injected and cardiovascular responses determined. The data were compared with the L-NAME group and expressed as mean ± SEM. One-way ANOVA followed by Tukey's post hoc test (*n* = 7) Notes: ***: *P* < 0.001 compared to the control; #: *P* < 0.05; ##: *P* < 0.01; ###: *P* < 0.001 compared to the L-NAME group; +: *P* < 0.05; ++: *P* < 0.01 compared to the SNP; $: *P* < 0.05 compared to the lower dose

The injection of L-NAME increased SBP, MAP and HR (Figure 1). The changes of HR, SBP and MAP after the injection of L-NAME are illustrated in Figure 2. As it is shown, ΔSBP, ΔMAP and ΔHR significantly increase in the L-NAME group (*P* < 0.05), compared to those reported for the control group (*P* < 0.001). The pre-treatment with SNP significantly attenuated cardiovascular responses induced by L-NAME (through the reduction of ΔSBP, ΔMAP and ΔHR [*P* < 0. 01]), in comparison to that in the L-NAME group.

### Effect of Pre-treatment with Aqueous Fractions of ZJ Extract on Cardiovascular Responses Induced by L-NAME

**Figure 3:**
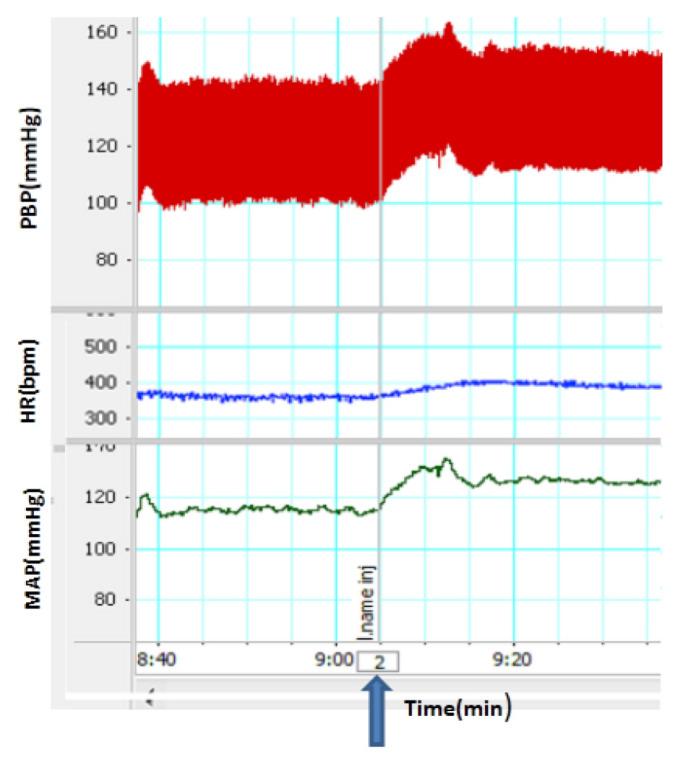
A recorded sample of the effect of pre-treatment with an aqueous fraction of ZJ on cardiovascular responses induced by L-NAME. The blue arrow indicates the time of injection

Two doses of aqueous fraction were used in this experiment. A 150 mg/kg dose of aqueous fraction significantly attenuated ΔSBP and ΔMAP induced by L-NAME (*P* < 0.001). The tachycardia induced by L-NAME also significantly decreased by this fraction (*P* < 0.05). With a 300 mg/kg dose of aqueous fraction, ΔSBP and ΔMAP also significantly reduced, compared to that of the L-NAME group (Figures 2 and 3; *P* < 0.01). The comparison of the aforementioned doses showed that the cardiovascular effects of the lower dose on ΔSBP, ΔMAP and ΔHR were significantly higher than those reported for the higher dose (*P* < 0.05).

### Effect of Pre-treatment with Ethyl Acetate Fractions of ZJ Extract on Cardiovascular Responses Induced by L-NAME

**Figure 4:**
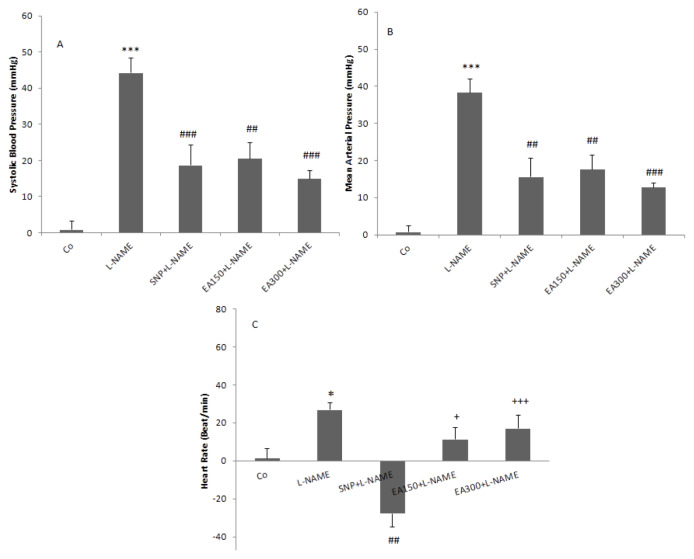
Effects of ethyl acetate fractions of the ZJ extract on ΔSBP (A), ΔMAP (B), and ΔHR(C) in L-NAME hypertensive rats. Two doses of the fraction (150 mg/kg and 300 mg/kg) (EA150 and EA300) administrated orally for four weeks then L-NAME injected and cardiovascular responses determined. The data were compared with the L-NAME group and expressed as mean ± SEM. One-way ANOVA followed by Tukey's post hoc test (*n* = 7) Notes:***: *P* < 0.001 compared to the control; #: *P* < 0.05; ##: *P* < 0.01; ###: *P* < 0.001 compared to the L-NAME group; +: *P* < 0.05; +++: *P* < 0.001 compared to the SNP

**Figure 5:**
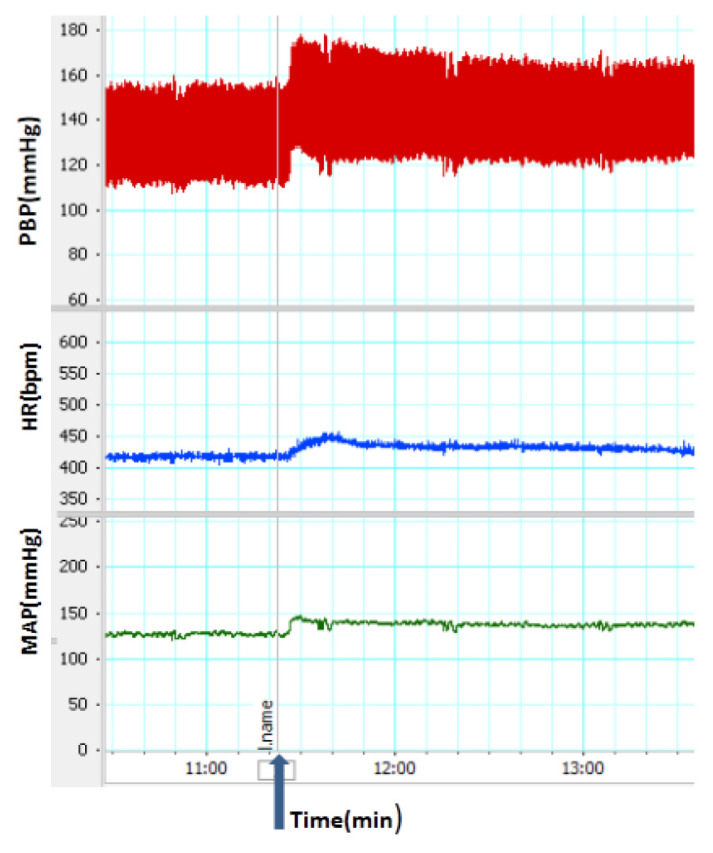
A recorded sample of the effect of pre-treatment with ethyl acetate fraction of ZJ on cardiovascular responses induced by L-NAME. The blue arrow indicates the time of injection

The effects of 150 mg/kg and 300 mg/ kg doses of ethyl acetate fractions on HTN induced by L-NAME were evaluated in this study. According to the obtained results, it was indicated that ethyl acetate 150 mg/kg significantly decreased ΔSBP and ΔMAP in comparison to L-NAME (*P* < 0.05 and *P* < 0.01). A 300 mg/kg dose of ethyl acetate also significantly reduced both ΔSBP and ΔMAP (*P* < 0.001), compared to that of the L-NAME group. In this fraction, the higher dose was more effective than the lower dose but with no significance. The ΔHR in both doses was lower than that reported for the L-NAME group but with no significance (*P* < 0.05) (Figures 4 and 5).

### Comparison of Aqueous and Ethyl Acetate Fractions of ZJ Extract on Cardiovascular Responses in L-NAMEInduced Hypertensive Rats

**Figure 6:**
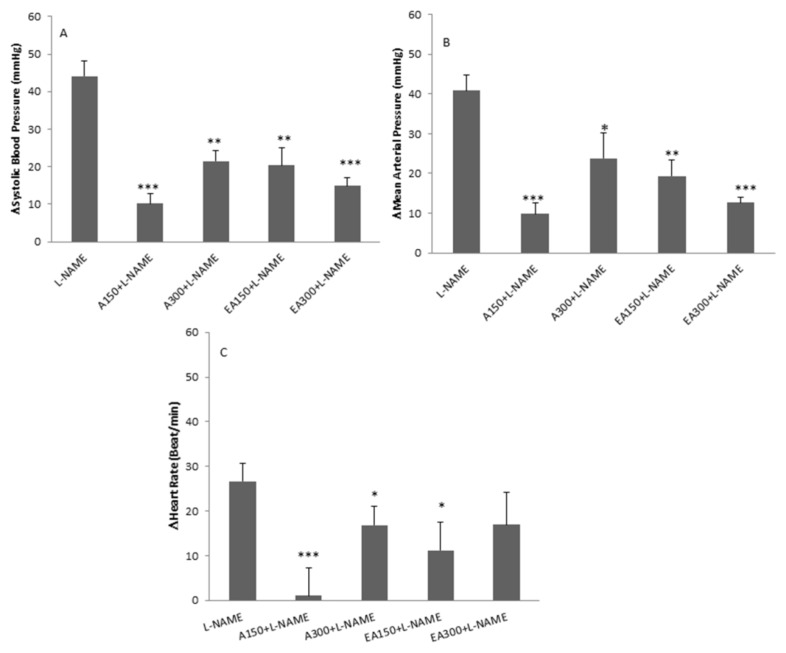
Comparison cardiovascular effect between different doses of both aqueous and ethyl acetate fractions of the ZJ extract in L-NAME hypertensive rats. The data were compared with the L-NAME group and expressed as mean ± SEM. One-way ANOVA followed by Tukey's post hoc test (*n* = 7) Notes: *: *P* < 0.05; **: *P* < 0.01; ***: *P* < 0.001 compared to the L-NAME group

Figure 6 illustrates the comparison of both doses of aqueous and ethyl acetate fractions of ZJ extract. As it is shown, both doses of fractions significantly ameliorate ΔSBP and ΔMAP induced by L-NAME (*P* < 0.05 to *P* < 0.001). However, a 150 mg/kg dose of the aqueous fraction was more effective than 300 mg/kg dose of aqueous and both doses of ethyl acetate fraction. The effect of 150 mg/kg dose of the aqueous fraction on HR induced by L-NAME was significant, compared to that reported for a 300 mg/kg dose of aqueous fraction and 150 mg/kg and 300 mg/kg doses of ethyl acetate.

## Discussion

The obtained results of this study showed that both aqueous and ethyl acetate fractions of ZJ had a beneficial effect on acute HTN induced by L-NAME; nevertheless, a lower dose of aqueous fraction and higher dose of ethyl acetate were more effective in the attenuation of cardiovascular parameters. The findings of the present study are associated with the results of a study carried out by Kim and Ham indicating that ZJ extract stimulates NO release in vitro and in vivo conditions (21). Therefore, both fractions may be involved in the release of NO in endothelium and decreased BP.

Better effect of the aqueous fraction than the ethyl acetate fraction could be attributed to the type of solvent used in fraction preparation. According to the literature, ZJ extract contains large amounts of polyphenols and flavonoids. Molecularly, flavonoids have two phenolic portions on their building, and their polarity is less than polyphenols, such as tannin. Accordingly, flavonoids and polyphenols have the best solubility in nonpolar and polar solvents, respectively. Therefore, the aqueous fraction of the ZJ extract due to a high polarisation of water molecules has the highest polyphenol compounds. Moreover, ethyl acetate fractions due to low polarity have the highest flavonoids. According to a previous study, it was shown that flavonoids and polyphenols have hypotensive effects similar to antioxidants (22).

In the current study, the aqueous fraction had an effect on L-NAME-induced HTN. Since polyphenols were present in aqueous fraction, it was proposed that the effect of the fraction was mediated by these compounds. In consistent with this finding, several studies showed that polyphenols directly interact with superoxide anions and other reactive oxygen species (ROS) agents, such as peroxy and hydroxyl radicals, and reduce their activity at the cellular and tissue levels. In other words, polyphenols play the role of free radical scavengers.

The strong sources of ROS in vessel cells are nicotinamide adenine dinucleotide phosphate (NADPH) oxidase, xanthine oxidase, and even uncoupled eNOS (23–25). Polyphenols inhibit the expression of NADPH oxidase and upregulate the expression and production of catalase (26). Because antioxidants improve HTN, this fraction via antioxidant properties may involve in the reduction of BP.

Some polyphenols and jujuboside activate eNOS via the stimulation of G protein-coupled receptors (GPCRs). The activation of GPCRs induces the endothelial NO generation via calcium/calmodulin and phosphatidylinositol 3-kinase/Akt signaling pathways with the SERIN_1177_ phosphorylation on the eNOS enzyme (27). In addition, the results of another study revealed that using flavonoids significantly increased platelet-derived NO release and decreased superoxide dismutase in vivo and in vitro conditions (28). According to the results of the aforementioned study, it can be concluded that flavonoids, in addition to antioxidant properties, have antihypertensive properties by increasing NO levels.

Another study concluded that flavonoids also prevent the reaction between NO and free radicals that produce NO radicals (29). Flavonoids also reduce plasma endothelin concentration and enhance NO status resulting in vasorelaxation and improvement of endothelial function (30). Moreover, polyphenols also influence HR probably via an effect on the heart β-adrenergic receptors (31).

Another important ingredient of ZJ extract is a glycoside called Jujuboside as previously mentioned. Molecularly, this compound (C_58_H_94_O_26_) is more soluble in medium-polar solvents, such as ethyl acetate, and its modulating effect on BP was confirmed in hypertensive rats based on the evidence (32). Jujuboside type B increases the influx of extracellular calcium ions through endothelial transient receptor potential cation channels, phosphorylates eNOS, and stimulates NO generation in vascular endothelial cells. Furthermore, vasodilation induced by Jujuboside B involves endothelium-dependent hyperpolarisation via potassium channels (33).

Among other groups of substances in the fractions of ZJ extract are saponins. Molecularly, they are divided into two groups of terpenoid and steroid, each of which has two hydrophilic and hydrophilic portions. Therefore, they have fairly good solubility in ethyl acetate solvent. These compounds have an inhibitory effect on NO release (34) and angiotensin-converting enzyme (35). Finally, because both fractions have a compound with a beneficial effect on BP, the present study suggested that the compounds of both fractions have a beneficial effect on the cardiovascular system.

## Conclusion

The results of the present study showed that aqueous and ethyl acetate fractions of ZJ prevented the development of HTN induced by L-NAME through the effect on NO bioavailability. Based on the obtained findings, the current study showed the beneficial effects of both fractions of ZJ as an anti-hypertensive agent on cardiovascular response.
